# Supplementary material for: Asynchronous magnetic resonance elastography: Shear wave speed reconstruction using noise correlation of incoherent waves
Source: Magn Reson Med. 2022 Oct 27;89(3):990–1001. doi: 10.1002/mrm.29502 (PMC9792433; doi:10.1002/mrm.29502)
Supplement: Supplementary file 1 — DATA S1 MATLAB code used to generate the numerical simulations of Figure 1 [file MRM-89-990-s002.zip › k-Wave/helpfiles/k-wave_license.html]

License (k-Wave)


# License

k-Wave © 2009-2020 Bradley Treeby, Ben Cox, and Jiri Jaros (see individual files for full list of authors).

The k-Wave toolbox is distributed by the copyright owners under the terms of the GNU Lesser General Public License (LGPL) which is a set of additional permissions added to the GNU General Public License (GPL). The full text of both licenses is included with the toolbox in the folder 'license'.

The license places copyleft restrictions on the k-Wave toolbox. Essentially, anyone can use the software for any purpose (commercial or non-commercial), the source code for the toolbox is freely available, and anyone can redistribute the software (in its original form or modified) as long as the distributed product comes with the full source code and is also licensed under the LGPL. You can make private modified versions of the toolbox without any obligation to divulge the modifications so long as the modified software is not distributed to anyone else. The copyleft restrictions only apply directly to the toolbox, but not to other (non-derivative) software that simply links to or uses the toolbox. k-Wave is distributed in the hope that it will be useful, but WITHOUT ANY WARRANTY; without even the implied warranty of MERCHANTABILITY or FITNESS FOR A PARTICULAR PURPOSE. See the GNU Lesser General Public License for more details.

If you find the toolbox useful for your academic work, please consider citing one or more of the following papers:

1. Overview of the toolbox with applications in photoacoustics:  
   B. E. Treeby and B. T. Cox, "k-Wave: MATLAB toolbox for the simulation and reconstruction of photoacoustic wave-fields," *J. Biomed. Opt.,* vol. 15, no. 2, p. 021314, 2010. online pdf- Nonlinear ultrasound model and the C++ code:  
     B. E. Treeby, J. Jaros, A. P. Rendell, and B. T. Cox, "Modeling nonlinear ultrasound propagation in heterogeneous media with power law absorption using a k-space pseudospectral method," *J. Acoust. Soc. Am.*, vol. 131, no. 6, pp. 4324-4336, 2012. online pdf
   - Elastic wave model:  
     B. E. Treeby, J. Jaros, D. Rohrbach, B. T. Cox , "Modelling elastic wave propagation using the k-Wave MATLAB toolbox," *IEEE International Ultrasonics Symposium*, pp. 146-149, 2014. online pdf
